# Effects of Co-Solvent-Induced Self-Assembled Graphene-PVDF Composite Film on Piezoelectric Application

**DOI:** 10.3390/polym15010137

**Published:** 2022-12-28

**Authors:** Januar Widakdo, Wen-Ching Lei, Anawati Anawati, Subrahmanya Thagare Manjunatha, Hannah Faye M. Austria, Owen Setiawan, Tsung-Han Huang, Yu-Hsuan Chiao, Wei-Song Hung, Ming-Hua Ho

**Affiliations:** 1 Advanced Membrane Materials Research Center, Graduate Institute of Applied Science and Technology, National Taiwan University of Science and Technology, Taipei 106335, Taiwan; 2 Department of Chemical Engineering, National Taiwan University of Science and Technology, Taipei 10617, Taiwan; 3 Department of Physics, Faculty of Mathematics and Natural Sciences, Universitas Indonesia, Depok 16424, Indonesia; 4 Research Center for Membrane and Film Technology, Kobe University, Kobe 657-8501, Japan; 5 Department of Chemical Science and Engineering, Kobe University, Kobe 657-8501, Japan

**Keywords:** PVDF, graphene, piezoelectric, composite film application, sensor

## Abstract

A persistent purpose for self-powered and wearable electronic devices is the fabrication of graphene-PVDF piezoelectric nanogenerators with various co-solvents that could provide enhanced levels of durability and stability while generating a higher output. This study resulted in a piezoelectric nanogenerator based on a composite film composed of graphene, and poly (vinylidene fluoride) (PVDF) as a flexible polymer matrix that delivers high performance, flexibility, and cost-effectiveness. By adjusting the co-solvent in the solution, a graphene-PVDF piezoelectric nanogenerator can be created (acetone, THF, water, and EtOH). The solution becomes less viscous and is more diluted the more significant the concentration of co-solvents, such as acetone, THF, and EtOH. Additionally, when the density is low, the thickness will be thinner. The final film thickness for all is ~25 µm. Furthermore, the- crystal phase becomes more apparent when graphene is added and combined with the four co-solvents. Based on the XRD results, the peak changes to the right, which can be inferred to be more dominant with the β-phase. THF is the co-solvent with the highest piezoelectric output among other co-solvents. Most of the output voltages produced are 0.071 V and are more significant than the rest.

## 1. Introduction

An attractive possibility for producing electricity is to capture mechanical energy from the vibrations in the surroundings that would otherwise go to waste. Piezoelectric [1], triboelectric [2,3], and other technologies can be used for mechanical energy harvesting. Due to its high output, ease of fabrication, and straightforward design, mechanical energy harvesting via the piezoelectric approach is particularly intriguing [4,5]. In addition, piezoelectric nanogenerators (PENGs) are adaptable, extremely effective, and mechanically durable tools that may supply electrical energy to autonomous systems [6,7]. A piezoelectric polymer called poly (vinylidene fluoride) (PVDF) is a prime candidate for use in mechanical energy harvesting based on the piezoelectric effect [8,9]. Mechanical strength, chemical stability, and biocompatibility are three of PVDF’s many benefits, making it a prime choice for mechanical energy harvesting by creating a piezoelectric nanogenerator (PENG) to supply electricity to electronic devices.

A polymer with a repeating unit of (CH_2_–CF_2_)_n_ is called PVDF [10,11]. It demonstrates muscular mechanical strength, excellent thermal stability, good chemical resistance, and excellent aging resistance, which are crucial for mechanical energy harvesting [12]. Additionally, PVDF has good processability to make flat sheets [13], hollow fiber sheets [14], or tubular composite films. The first three of the four crystalline phases of PVDF α, β, γ, and ε phase are the most prevalent [15]. The most frequent crystal structure that develops when PVDF is processed in a solution is the α-phase. Compared to other crystal phases, the β-phase, which possesses a trans-planar zigzag structure (TTTT), results in the most extensive spontaneous polarization per unit cell, which helps explain the substance’s remarkable pyroelectric and piezoelectric capabilities [16]. The γ-phase (TTGTTTGT) exhibited may be produced by melt-crystallization and solution processes at temperatures higher than 160 °C [17]. The most desirable form of PVDF crystal among these crystalline phases is the β-phase, which has the greatest ferroelectric and piezoelectric capabilities and the most significant spontaneous polarization, and may be used in various ways [18]. Therefore, the investigation and fabrication of the β-phase PVDF have received much attention.

In addition, several previous studies have looked at adding other materials to increase further the electrical behavior of PVDF, including PVDF-TrFE [19], carbon nanotubes (CNTs) [20], graphite-carbon nano-layers (CNS)-CNT [21], BaTiO_3_-piezoelectric nanoparticles (NPs) [22], polytetrafluoroethylene-copper (PTFE-Cu) [21], Fe_3_O_4_-graphene oxide/hybrid nanocomposites [23], graphene oxide (GO), and graphene [14,16,24]. As electrodes for PVDF devices, a few flexible conducting materials have been used, with graphene receiving the most attention [14,16,25,26,27]. Due to its exceptional physical qualities, including high electrical conductivity, high thermal conductivity, and optical transparency, graphene has been investigated as a transparent electrode material [28,29]. Graphene has been proposed as a replacement for indium tin oxide thin film for a transparent electrode. Due to its flexibility and stretchability, numerous research groups have recently employed graphene as electrodes for energy-harvesting devices [30,31].

More than adding other materials is needed to increase the piezoelectric properties of PVDF. There are previous studies that have examined the effects of solvent and cosolvent on polymeric PVDF/graphene films; for example, using NMP [32,33], DMF [34], DMF/water [35], and DMF/acetone [36]. However, some solvents have problems combining PVDF and graphene due to their high viscosity, which causes the solution to be inhomogeneous. Various strategies have been developed to induce the β-phase in PVDF. The β-phase in PVDF can be obtained by various strategies, such as shear [37], uniaxial stretching [38], rolling [39], polling under a high electric field [40], and mixing with a different solvent [41]. The common solvents *N*,*N*-dimethylacetamide (DMAc) [42], dimethylformamide (DMF) [43], and *N*-methyl-2-pyrrolidone (NMP) [26,44] are soluble in PVDF. The cosolvent approach helped to disperse graphene in a high aspect ratio in the PVDF matrix. Dielectric results showed that adding graphene increased the electrical conductivity of the composites.

In this study, we constructed a piezoelectric generator using a PVDF film that contained a combination of graphene. We incorporate particular co-solvents into the NMP solution, such as acetone, tetrahydrofuran (THF), water, and EtOH. The spherulitic size of the PVDF was reported to have decreased, and the crystallization kinetics to have been affected by the addition of a co-solvent. The nucleation of the PVDF phase was also shown to be more successfully promoted by graphene with a negative surface charge. Two other efficient ways to boost the piezoelectric performance of PVDF nanofibers for energy harvesting applications include using polar solvents to dissolve and recrystallize the material, and adding additions such as graphene.

## 2. Materials and Methods

### 2.1. Materials

Polyvinylidene fluoride (PVDF, Polyvinylidene fluoride, Aldrich, Saint-Louis, MO, USA), *N*-methyl pyrrolidone (NMP, 1-Methyl-2-pyrrolidone, UniRegion Bio-Tech, Hsinchu, Taiwan), and acetone were acquired from Echo Chemical Co., Ltd. Taiwan, China; tetrahydrofuran (THF, Tetrahydrofuran, 99.9%, DUKSAN/Macron) and ethanol (EtOH was acquired from Echo Chemical Co., Ltd. Taiwan, China); graphene (Graphene, UC Bacon Company Ltd., Taoyuan, Taiwan), DI water, and a glass plate.

### 2.2. Mixing Different Co-Solvents with PVDF

Making 10 wt% water/*N*-methyl pyrrolidones (NMP), the first thing to do is mix 5.4 g of water and 48.6 g of *N*-methyl pyrrolidone. Stir well with ultrasonic vibration for 30 min. Next, take 10 g of the mixed solvent and add 6 g of polyvinylidene fluoride (PVDF). Heat all the solutions and stir until dissolved using a magnetic hotplate stirrer. Repeat the above steps by replacing the aqueous solution with different co-solvents, such as acetone, tetrahydrofuran (THF), and EtOH in the NMP solution.

### 2.3. Fabrication of Graphene–PVDF Composite Film with Different Co-Solvent

**Figure 1:**
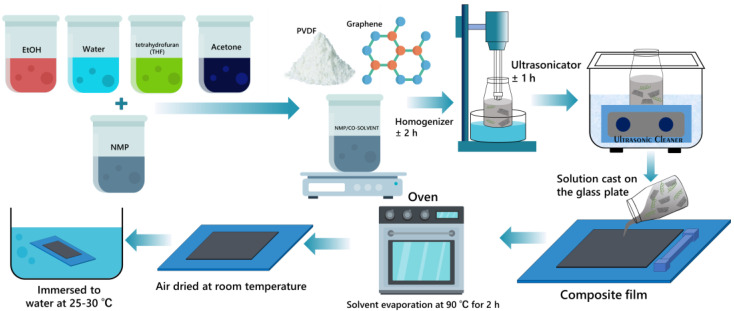
Schematic preparation of graphene–PVDF piezoelectric nanogenerator composite film with different co–solvents.

Using a dry technique, the PVDF–graphene composite film was created (Figure 1). In a nutshell, a rotor-stator homogenizer (125 Ultra-Turrax, IKA Works Inc., Wilmington, CN, USA) was used to agitate a mixture of powders containing ten 10 wt% graphene and 10 wt% PVDF at a speed of 8000 rpm for 2 h. The resulting homogenized solution was put through an ultrasonic degassing process at room temperature for an hour. The degassed solution was then cast on a glass substrate while keeping a 0.1 mm distance between the casting knife’s surface and the glass substrate; then, it was dried at 90 °C for one hour. The composite film was removed from the glass substrate after drying. The room temperature and humidity for the composite film casting were 24 °C and 67.1%, respectively.

### 2.4. Characterization of the Graphene–PVDF Composite Film with Different Co-Solvent

To study the surface composition of the composite film, the composites were characterized using attenuated total reflectance—Fourier transform infrared spectroscopy (ATR-FTIR) (PerkinElmer, Miracle-Dou, Waltham, MA, USA) and Raman spectroscopy (JASCO, NRS-5100, Tokyo, Japan). For morphological and microstructural studies, scanning electron microscopy (SEM) (JEOL, JSM-6900LV, Akishima, Japan) and X-ray powder diffraction analysis (Bruker, D2 PHASER XE-T XRD, Bremen, Germany) were carried out. Water contact angle (WCA) (Data Physics, OCA 25, Filderstadt, Germany) measurements were conducted to evaluate the composite film surface hydrophilicity or hydrophobicity. The viscosity of graphene–PVDF solutions with different cosolvent concentrations (ranging from 10 to 40 wt%) was measured at 25 °C using an SV-100 Vibration Viscometer (A&D Company, Ltd. Tokyo, Japan).

### 2.5. Piezoelectric Test of the Graphene–PVDF Composite Film with Different Co-Solvent

In this study, the piezoelectric performance of the composite film will be investigated by applying the reciprocating 1 Hz impact. PVDF/graphene will be cut into 5 cm × 8 cm and placed in front of a linear motor after attaching the electrode. The charge will be generated to the two side surfaces of the composite film due to the piezoelectric effect, and was measured by connecting the composite film to the charge amplifier and dynamic signal acquisition module (National Instruments, NI-9234, Cary, NC, USA).

## 3. Results and Discussion

### 3.1. Photograph of Dispersion and Physical Characterization of Graphene–PVDF with Different Co-Solvent

**Figure 2:**
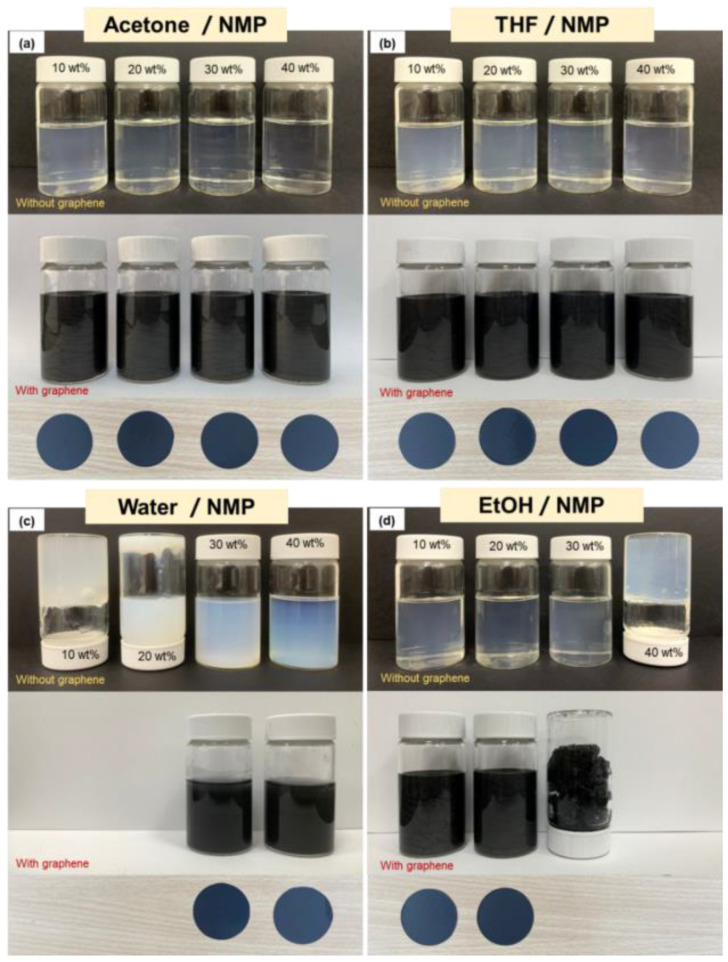
(**a**) Acetone/NMP; (**b**) THF/NMP; (**c**) water/NMP; (**d**) EtOH/NMP mixed solvent with PVDF and PVDF/graphene, the co-solvent concentration (acetone, THF, water, and EtOH) is 10 wt%, 20 wt%, 30 wt%, and 40 wt% from left to right.

Generally, the fabricated graphene–PVDF composite film morphology and topology depend on the solubility properties of the solvent and nonsolvent. Figure 2a–d shows a photograph of PVDF and graphene–PVDF with different co-solvent mixtures (acetone, THF, water, and EtOH). It can be seen from the picture above that in the mixed acetone/NMP and THF/NMP solutions, the higher the concentration of the co-solvent varied (from 10 to 40 wt%), the solution looked clearer and thinner. However, in co-solvent water, at a concentration of 10 and 20 wt%, the answer becomes viscous and forms a gel. As shown in Figure 2c, for 10 and 20 wt% water in the NMP/PVDF solutions, the lower the water concentration, the solution becomes a gel-like phase. In contrast to water, the EtOH co-solvent changes to a gel-like stage when the concentration is increased to 40 wt% (Figure 2d).

**Figure 3:**
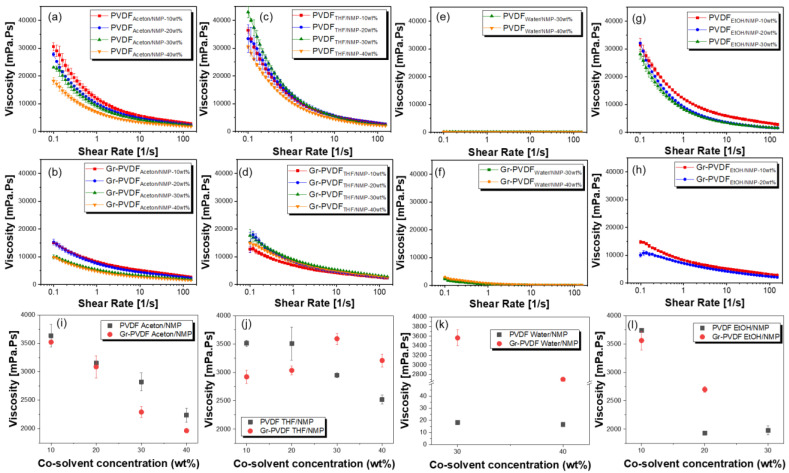
Viscosity changes upon the shear rate of (**a**) PVDF_Acetone/NMP_, (**b**) Gr-PVDF_Acetone/NMP_, (**c**) PVDF_THF/NMP_, (**d**) Gr-PVDF_THF/NMP_, (**e**) PVDF_water/NMP_, (**f**) Gr-PVDF_water/NMP_, (**g**) PVDF_EtOH/NMP_, (**h**) Gr-PVDF_EtOH/NMP_ solutions with different co-polymer concentrations. (**i**–**l**) Apparent viscosity at a shear rate of 60 s^−1^.

To prove the viscosity of a solution mixed with graphene, we carried out a viscosity test. The results of the viscosity test are shown in Figure 3. Figure 3a–d,g,h show the difference in the co-solvent concentration, namely acetone, THF, and EtOH (10 wt% to 40 wt%) with and without graphene. As the co-solvent concentration increases, the solution becomes more dilute; thus, the viscosity automatically decreases. When adding graphene, the viscosity decreases compared to before adding graphene because graphene has lubricant-like properties (which will be explained next from the Raman spectroscopy results). In addition, the viscosity mixed with co-solvent water is very low, even though graphene was added to the solution (Figure 3e,f).

Figure 3i–l shows that with an increase in viscosity, the phase inversion process becomes slow. Therefore, during phase inversion, the polymer chains have enough time to align slowly and form a highly crystalline phase so that the film will become dense, which improves piezoelectric performance in the presence of a graphene filler. With the decrease of viscosity, solvent–nonsolvent de-mixing will be fast; thus, the result and polymer phase will be less crystalline or may contain some pores. Therefore, the optimum viscosity of the solution is essential to prepare better films.

### 3.2. Morphology of the Piezoelectric Nanogenerators Graphene–PVDF Composite Film

**Figure 4:**
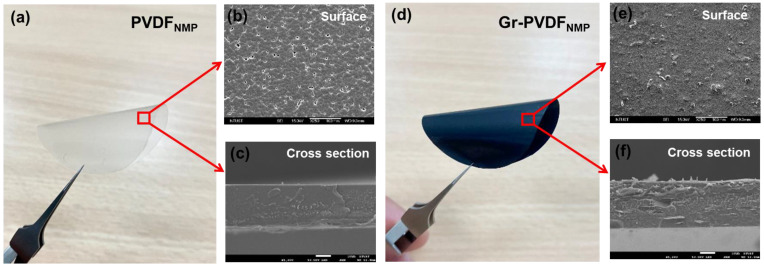
(**a**) Photograph of PVDF/NMP composite film. (**b**) PVDF_NMP_ SEM surface. (**c**) The PVDF_NMP_ SEM cross-section. (**d**) Photograph of Gr-PVDF_NMP_ composite film. (**e**) Gr-PVDF_NMP_ surface. (**f**) Cross-section of Gr-PVDF_NMP_.

For the use of piezoelectricity, the microstructure is essential. It is not easy to evenly distribute much graphene in a PVDF composite sheet. With a 10 wt% graphene load in a PVDF composite film, we demonstrate in this study how to use a powerful shear force to overcome a challenge that was once unsurmountable. Photographs of the PVDF and graphene–PVDF composite films are shown in Figure 4. The pristine PVDF composite film appears to have a dense structure in the cross-section and a slightly porous surface (Figure 4a–c). Additionally, when graphene is added, the transparent PVDF composite film turns black, and the composite films maintain their flexibility (Figure 4d). The composite film surface has graphene flakes that resemble fish scales as the graphene content increases, as seen in the scanning electron microscope (SEM) pictures (Figure 4e). The anticipated phase transition of the composite graphene–PVDF film is seen in the cross-sectional SEM images (Figure 4f).

**Figure 5:**
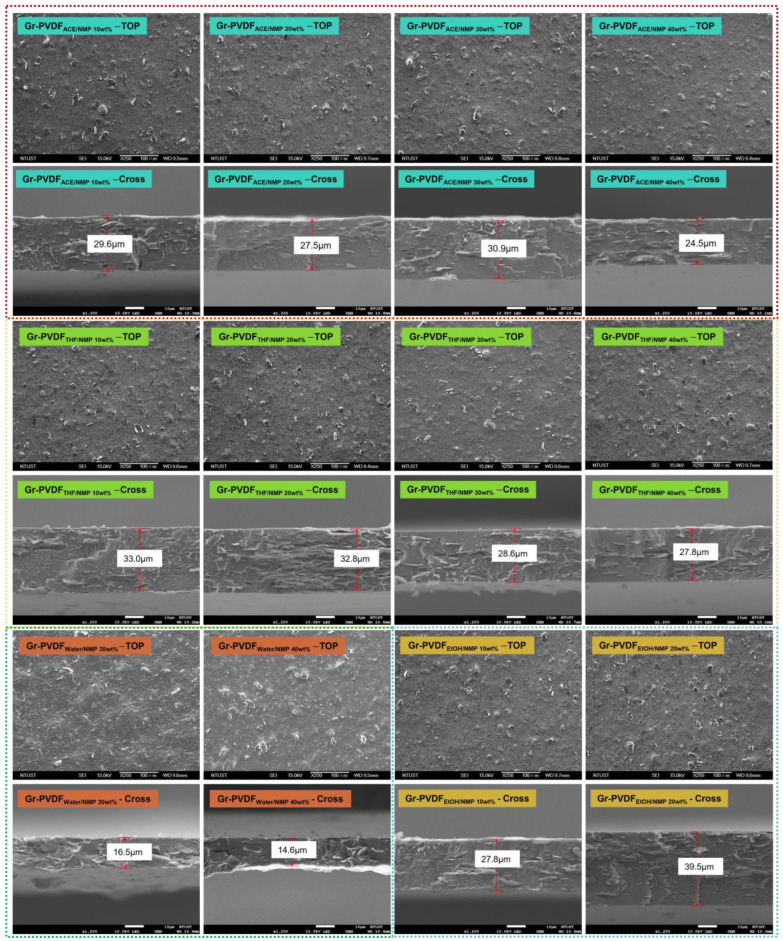
SEM of cross-section and the top surface of the composite film Gr-PVDF with different solvents (acetone/NMP, THF/NMP, water/NMP, and EtOH/NMP): the top surface with the magnification × 2500 and the part cross-section with the magnification × 1200.

The following data are the SEM characterization of graphene–PVDF with co-solvent variations. In Figure 5, the surface morphology and cross-section are shown. All the surface morphologies look rough, and it can be seen that graphene powder appears on the surface of the composite film compared to the pristine PVDF morphology (Figure 4b). Those show that graphene was mixed evenly in the PVDF solution. Graphene–PVDF composite films are hydrophobic with a water contact angle of ~90° (Figure S1, see Supplementary Materials). The rough surface will affect the hydrophilicity and hydrophobicity properties of the composite film. When the surface film gets rougher, the film will have superhydrophobic properties. The hydrophobic nature of the composite film will provide benefits to be able to have self-cleaning capabilities; for example, when the composite film is exposed to dust or dirt. Then, the composite film can be easily cleaned using water. The composite film has hydrophobic properties in this study, as shown in the water contact angle results (Figure S1, see Supplementary Materials). Therefore, the benefits of having a rough surface are as above.

Furthermore, in relation to graphene–PVDF with a mixture of NMP solvent and co-solvent acetone, the cross-section will decrease with the increasing concentration of the co-solvent. This trend is also shown in the co-solvent THF and EtOH. Solvent/co-solvent mixtures such as NMP/acetone and NMP/THF show that the viscosity will decrease or become thinner when the co-solvent concentration increases. Therefore, the dilute mixture will be easier to spread on the glass plate, causing the thickness of the composite film to decrease and become thinner. Co-solvent, polymeric additive, polymer, and solvent will influence the composite film thickness and viscosity change for a given system [45].

### 3.3. Chemical Characterization of the Graphene–PVDF Composite Film with Different Co-Solvent

**Figure 6:**
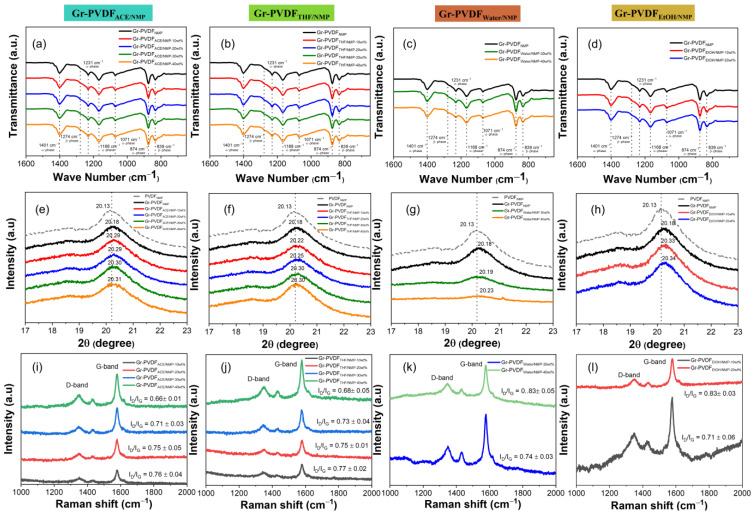
(**a**–**d**) FTIR-ATR spectra, (**e**–**h**) X-Ray diffraction, (**i**–**l**) Raman spectroscopy of the PVDF composite film’s top surface after it was made with several co-solvents.

Since the crystallization environment differs depending on the sample thickness, variations in crystalline structure may be present. Attenuated total reflectance (ATR) is the most widely used sampling methodology for Fourier transform infrared (FTIR) spectroscopy. The FTIR-ATR spectroscopy method was used to examine the crystalline structure of the composite film top surfaces. Between 1600 to 650 cm^−1^, the penetration depth ranges from 2.00–2.86 μm. The top surface’s crystalline structure mostly matches the bulks for all the graphene–PVDF composite films, as seen in Figure 6a–d. While the bands at 839 cm^−1^ and 1274 cm^−1^ were attributed to the β-phase structure, the transmittance bands that characterize the pure PVDF at 874, 1071, 1168, 1231, and 1041 cm^−1^ correspond to the α-phase. According to a report, higher polymer concentration favored a better-orientated packing of CH_2_–CF_2_ dipoles and the TTTT confirmation of the β-phase that followed. When the graphene content was barely 10 wt%, almost all the β-phase was generated.

The XRD data (17 to 23 degrees) of the final composite films cast from various co-solvent systems are shown in Figure 6e–h. Diffraction peaks can be seen in all composite films at 2θ = 20.20°. According to reports, the orthorhombic β crystal phase’s distinctive peaks were located around 20.20, connected to the superimposition of diffraction planes of (1 1 0). All composite films from mixed co-solvents primarily consist of the β crystal phase. As the weight percentage of co-solvent (acetone, THF, water, and EtOH) increases, the β-crystal phase shifts to a higher theta. From this, it can be concluded that the graphene–PVDF composite film will have the crystal structure in the newly studied composite films predominantly comprising β-phases. However, specifically for co-solvent water, as the concentration of water increases, the crystallinity decreases and becomes more amorphous (Figure 6g). This is indicated that in the high concentration of water, the crystallinity of the polymer obviously decreases, which leads to a reduction of the piezoelectric performance, to be discussed in the following section. The graphene characteristic peak is at a 2θ angle of ~26.4° (002) (Figure S2, see Supplementary Materials).

Raman spectroscopy was used to further study the interfacial interactions occurring in the nanocomposites to support the data obtained from FTIR. Figure 6i–l indicates that graphene–PVDF exhibits two bands: the G band at ∼1580 cm^−1^ and the D band at ∼1355 cm^−1^, which are caused by disordered graphitic structures in sp2 hybridized carbons, respectively. The ID/IG ratios in the figure show where the D and G bands are located. The greater strength of the D bands compared to the G bands suggests that the disordered phase of graphene is increasing. The average cluster size, sp2, and the ID/IG ratio, which quantifies the degree of disorder, are inversely correlated [46]. The ID/IG ratios of graphene–PVDF with a high co-solvent concentration were lower than those without a co-solvent, as shown in Figure 6i–l. Those imply that additional or new graphitic domains are created, increasing the number of sp2 clusters. Additionally, those likewise showed a notable drop in graphene flaws. The lubricant-like characteristics of graphene, which support the reinforcement of composite sheets with excellent uniformity and flexibility, may be responsible for this reduction in defects [14].

### 3.4. Piezoelectric Test of Graphene–PVDF Composite Film with Different Co-Solvent

**Figure 7:**
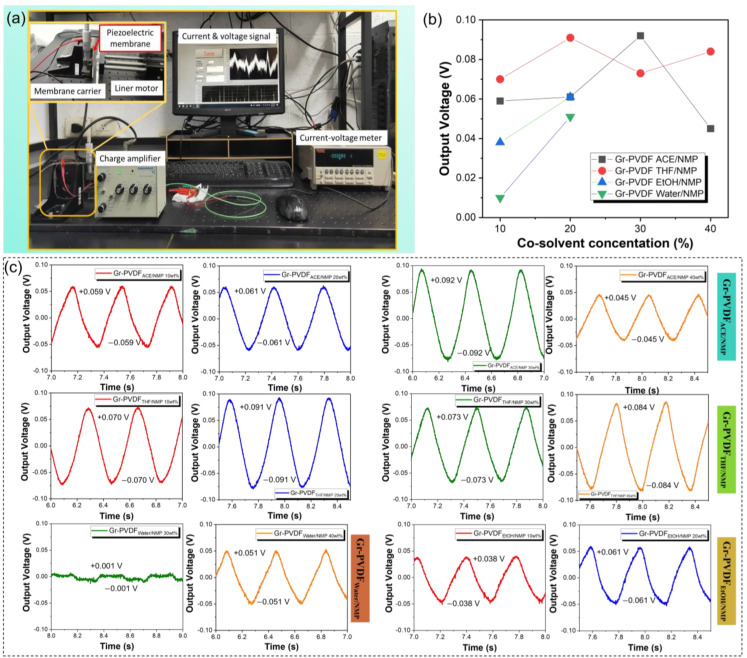
(**a**) Piezoelectric set-up instrument, (**b**) piezoelectric value (volt) of all composite film, and (**c**) the output voltage of graphene–PVDF piezoelectric nanogenerators composite film with different co-solvent at the input frequency of 1 Hz.

The press-hold-release method was used to investigate the conversion of mechanical energy to electrical for a liner motor action to show how the produced graphene–PVDF may be used as piezoelectric nanogenerators (Figure 7a). The output voltage of the graphene–PVDF piezoelectric film in NMP is shown in Figure 7b,c with various co-solvents (acetone, THF, water, and EtOH). All of the solutions contain 10 wt% of graphenes. In previous research, it has been tested that pristine PVDF is not conductive and does not have an output voltage. However, when varying the graphene concentration from 0 to 10 wt%, the output voltage increases to about 0.8 V (output electrical signals obtained using servo-hydraulic) when the graphene has a concentration of 10 wt% and when applied over a water wave [26].

In this study, we wanted to see the effect of adding co-solvent in the PVDF/Graphene/NMP solution. In the co-solvent acetone, the average output voltage is about 0.059 V. At the same time. THF was higher, at about 0.073 V, followed by EtOH at 0.061 V. However, the co-solvent water had a poor piezoelectric yield. Because the mixture of water/NMP causes the viscosity and graphene dispersion in the solution, which it needs to be better, all the co-solvent combinations increase the output voltage compared to before being given a co-solvent. The generated voltage is only 0.048 V (Figure S3, see Supplementary Materials). Therefore, we found that the THF co-solvent had a good effect on the piezoelectric performance when combined with the NMP solvent, PVDF polymer, and graphene.

We discovered that liner motor motions are well-synchronized using graphene–PVDF nanogenerators. The mechanical strain caused by the pressing movement along the thickness of the composite film necessitates the installation of an external circuit to carry free electrons across the positive and negative electrodes. These free electrons will accumulate at the electrode mixed film contact and create a positive voltage peak. The piezo potential balances free electrons via an external circuit during the holding action. No voltage peak was observed. However, during the release action, the piezo potential vanishes, and the accumulated free electrons are sucked back into the electrode, causing the observation of a negative signal that is typical of the activity of composite film generators.

Table 1 compares various PVDF piezoelectric films to the resulting output voltage. The table shows several variations of adding material to PVDF and the fabrication method. PVDF film fabrication using the electrospinning method has significant results compared to other methods; however, the electrospinning method uses energy that is not efficient. This research uses mixing fabrication by adding only graphene to the PVDF mixture. However, the results needed to be more significant even though the solution had been modified using various co-solvents. The researcher suggests a further review of other co-solvent materials and different fabrication methods.

**Figure 8:**
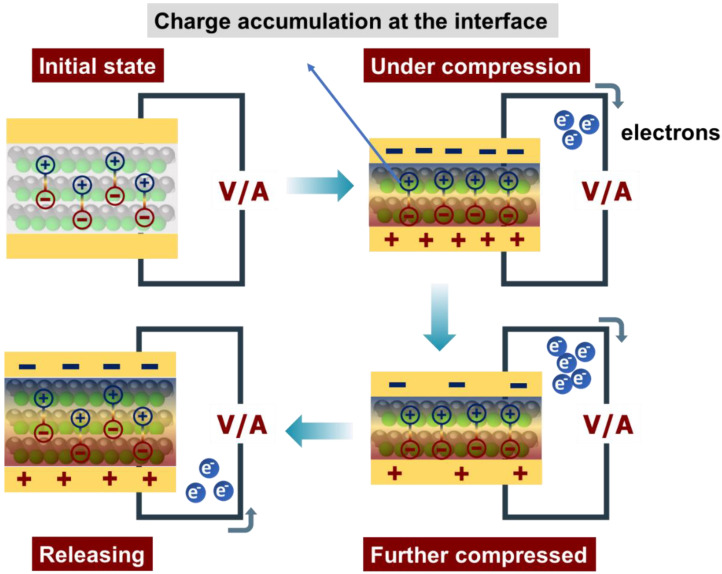
Working mechanisms of the piezoelectric composite film with a schematic illustration of the piezoelectric charge generation process.

In the early stage, as shown in Figure 8, no charges accumulated on the surface of the graphene–PVDF composite film. The filled graphene forms macroscopic dipoles due to the piezoelectric potential created in the crystalline PVDF when a force is applied to the graphene–PVDF piezoelectric nanogenerators. Charges with the opposite polarity then accumulate at the interface and surface of the graphene–PVDF composite. Due to the power being used, the lower charge density of the top and bottom electrodes permits charges to flow from the bottom electrode to the top electrode, producing a positive signal. As the charge density on the two electrodes increases during the release process, charges flow from the top electrode to the bottom electrode, producing a negative signal [52].

## 4. Conclusions

This research created a graphene–PVDF piezoelectric nanogenerator by varying the co-solvent in the solution (acetone, THF, water, and EtOH). The higher the co-solvent acetone, THF, and EtOH concentration, the lower the resulting viscosity and the more dilute the solution. In addition, the thickness will be thinner when the density is low. For all, the resulting composite film thickness is ~25 μm. Furthermore, the β-crystal phase is more evident when graphene is added and mixed with the four co-solvents. Judging from the XRD results, the peak β-phase shifts to the right, which can be assumed to be more dominant with the β-phase. Of the four co-solvents used, THF has a significant piezoelectric output. Almost all of the resulting output voltages are 0.071 V and more significant than the others.

## Figures and Tables

**Table 1:** Comparison of various types of PVDF piezoelectric films to the resulting output voltage.

Sample Name	Fabrication	Output Voltage (V)	Reference
PVDF-TrFE	Mixing	0.71	[47]
CNT–COOH/PVDF-T 1 wt%		1.22	
CNT–COOH:SMA EF40-M/PVDF-T 1:1		1.34	
PVDF S.NWs + AAO	Electrospinning	1.97	[48]
Ni NWs/PVDF	Electrospinning	1.75	[49]
PVDF/TiO2–Fe3O4–MWCNT	Electrospinning	51.42 mV/N	[50]
Fe–ZnO/PVDF	Gamma irradiation of casted films	2.4	[51]
Gr-PVDF_ACE/NMP 30 wt%_	Mixing	0.092	This work
Gr-PVDF_THF/NMP 20 wt%_	Mixing	0.091	
Gr-PVDF_water/NMP 40 wt%_	Mixing	0.051	
Gr-PVDF_EtOH/NMP 20 wt%_	Mixing	0.061	

## Data Availability

All data generated or analyzed during this study are included in this published article (and its supplementary information files).

## Supplementary Materials

The following supporting information can be downloaded at: https://www.mdpi.com/article/10.3390/polym15010137/s1, Figure S1: Water contact angle of graphene–PVDF piezoelectric nanogenerators membrane with different co-solvent; Figure S2: X-Ray diffraction of graphene–PVDF piezoelectric nanogenerators membrane with different co-solvent in a range of 23° to 30°; Figure S3: The output voltage of graphene–PVDF piezoelectric nanogenerators membrane.
