# Supplementary material for: Effects of Co-Solvent-Induced Self-Assembled Graphene-PVDF Composite Film on Piezoelectric Application
Source: Polymers (Basel). 2022 Dec 28;15(1):137. doi: 10.3390/polym15010137 (PMC9824748; doi:10.3390/polym15010137)
Supplement: Supplementary file 1 [file polymers-15-00137-s001.zip › polymers-2081508-supplementary.pdf]

Supplementary Information: Effects of Co-Solvent-Induced Self-Assembled Graphene-PVDF Composite Film on Piezoelectric Application

Januar Widakdo [1,3], Wen-Ching Lei [2], Anawati Anawati [3], Subrahmanya Thagare Manjunatha [1], Hannah Faye M. Austria [1], Owen Setiawan [1], Tsung-Han Huang [1], Yu-Hsuan Chiao [4,5,*], Wei-Song Hung [1,*] and Ming-Hua Ho [2,*]

[1] Advanced Membrane Materials Research Center, Graduate Institute of Applied Science and Technology, National Taiwan University of Science and Technology, Taipei 106335, Taiwan

[2] Department of Chemical Engineering, National Taiwan University of Science and Technology, Taipei 10617, Taiwan

[3] Department of Physics, Faculty of Mathematics and Natural Sciences, Universitas Indonesia, Depok 16424, Indonesia

[4] Research Center for Membrane and Film Technology, Kobe University, Kobe 657-8501, Japan

[5] Department of Chemical Science and Engineering, Kobe University, Kobe 657-8501, Japan

[*] Correspondence: ychiao@people.kobe-u.ac.jp (Y.-H.C.); wshung@mail.ntust.edu.tw (W.-S.H.); mhho@mail.ntust.edu.tw (M.-H.H.)

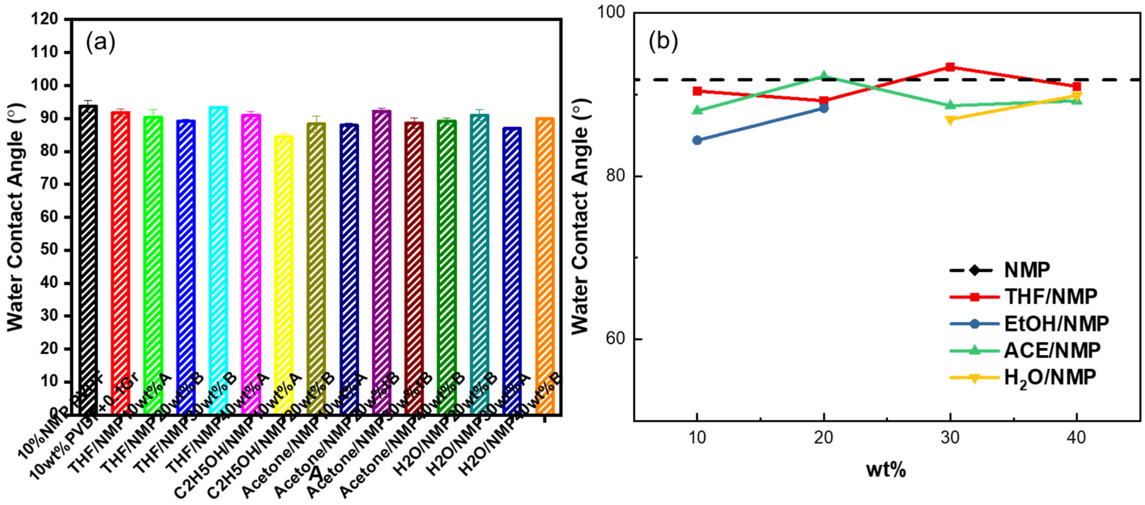

Figure S1. Water contact angle (a) histogram and (b) line data of composite film Gr-PVDF with different solvents acetone/NMP, THF/NMP, water/NMP, and EtOH/NMP.

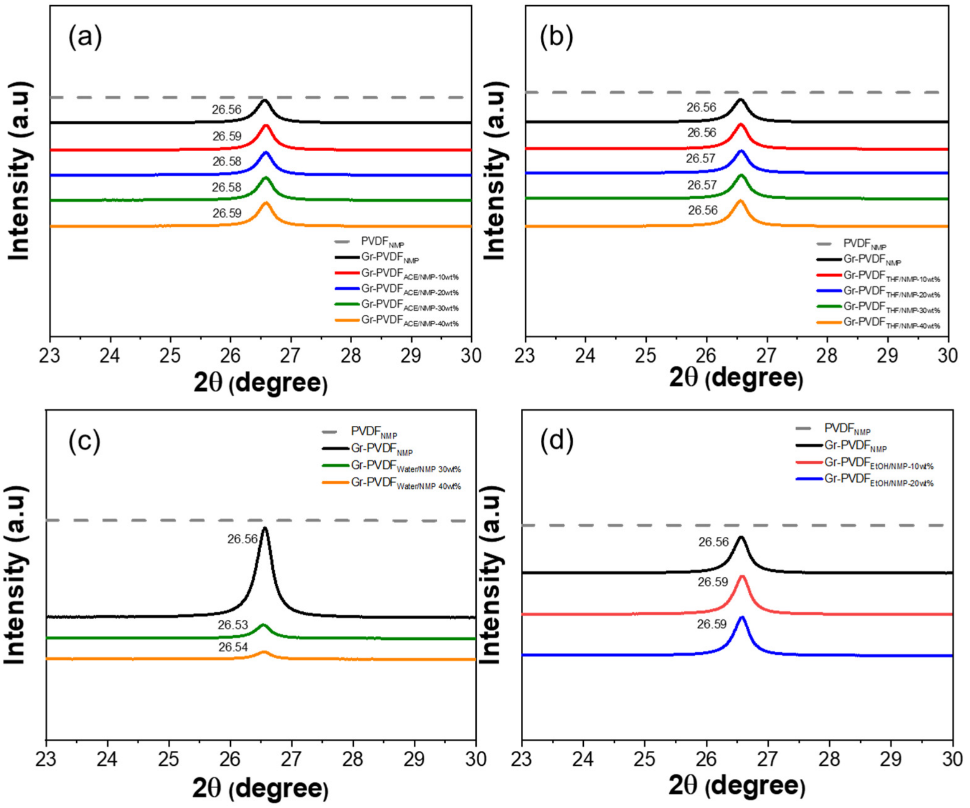

Figure S2. X-Ray diffraction of graphene-PVDF piezoelectric nanogenerators membrane with different co-solvent in a range of 23° to 30°. The composite film Gr-PVDF with different solvents (a) acetone/NMP, (b) THF/NMP, (c) water/NMP, and (d) EtOH/NMP.

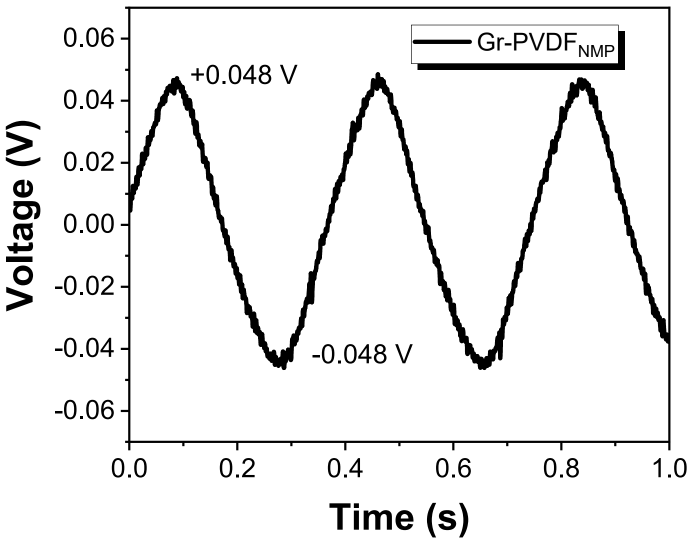

Figure S3. The output voltage of graphene-PVDF piezoelectric nanogenerators membrane.
